# Supplementary material for: Antimicrobial and antibiofilm activity of specialized metabolites isolated from Centaurea hyalolepis
Source: PeerJ. 2024 Mar 26;12:e16973. doi: 10.7717/peerj.16973 (PMC10979744; doi:10.7717/peerj.16973)
Supplement: Supplemental Information 1 [file peerj-12-16973-s001.docx]

**SUPPORTING INFORMATION**

Article

**Antimicrobial and Antibiofilm Activity of Specialized Metabolites Isolated from *Centaurea hyalolepis***

Shurooq Ismail 1,2, Marco Masi 1, Rosa Gaglione 1,3, Angela Arciello 1,3,*, Alessio Cimmino 1,*

^1^ Department of Chemical Sciences, University of Naples Federico II, Via Cintia 21, I-80126, Naples, Italy; [shurooq.ismail@unina.it](mailto:shurooq.ismail@unina.it) (S.I.); [marco.masi@unina.it](mailto:marco.masi@unina.it) (M.M.); [rosa.gaglione@unina.it](mailto:rosa.gaglione@unina.it) (R.G.); [anarciel@unina.it](mailto:anarciel@unina.it) (A.A.), [alessio.cimmino@unina.it](mailto:alessio.cimmino@unina.it) (A.C.)

^2^ Department of Biomedical Sciences, An-Najah National University, Nablus 97300, Palestine;

[shurooq.ismail@najah.edu](mailto:shurooq.ismail@najah.edu) (S.I.)

^3^ Istituto Nazionale di Biostrutture e Biosistemi (INBB), Rome, Italy

* Corresponding Authors:

Angela Arciello (A.A.), Alessio Cimmino (A.C.)

Via Cintia 21, I-80126, Naples, Italy

Email address: [anarciel@unina.it](mailto:anarciel@unina.it) (A.A.), [alessio.cimmino@unina.it](mailto:alessio.cimmino@unina.it) (A.C.)

**Supporting information list**

**Figure S1**: Calibration curve of Bradford assay determined by using a standard glucose (0-2 mg/mL) calibration curve.

**Figure S2**: Calibration curve of polysaccharide content of biofilms determined by using a standard glucose (0-5 mg/mL) calibration curve.

**Figure S3.** ^1^H NMR spectrum of cnicin (compound **1**) recorded in CDCl_3_ at 500 MHz.

**Figure S4.** ESI MS spectrum of cnicin (compound **1**) recorded in positive modality.

**Figure S5.** ^1^H NMR spectrum of 11β,13- dihydrosalonitenolide (compound **2**) recorded in CDCl_3_ at 500 MHz.

**Figure S6.** ESI MS spectrum of 11β,13-dihydrosalonitenolide (compound **2**) recorded in positive modality.

**Figure S7**. ^1^H NMR spectrum of salonitenolide (compound **3**) recorded in CD_3_OD at 500 MHz.

**Figure S8.** ESI MS spectrum of salonitenolide (compound **3**) recorded in positive modality.

**Table S1:** MIC values range of the tested bacterial strains against some antibitics.


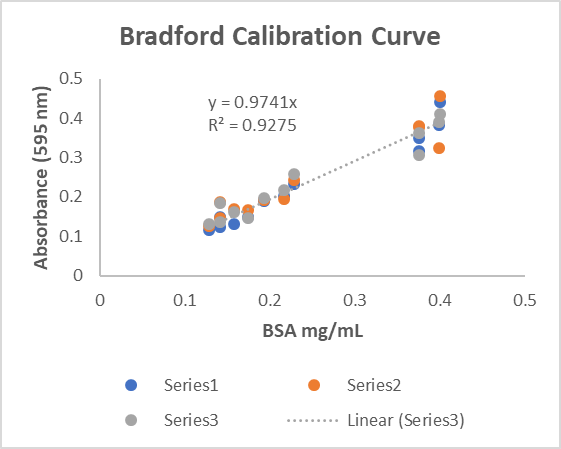


**Figure S1**: Calibration curve of Bradford assay determined by using a standard glucose (0-2 mg/mL) calibration curve. Data represents the mean (±standard deviation, SD) of at least three independent experiments.


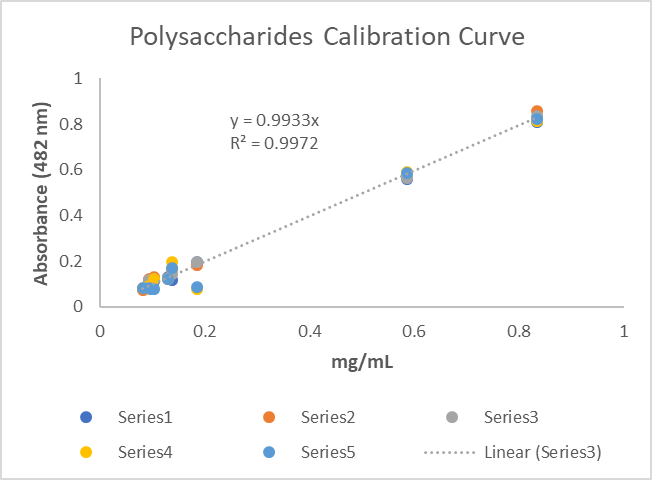


**Figure S2**: Calibration curve of polysaccharide content of biofilms determined by using a standard glucose (0-5 mg/mL) calibration curve. Data represents the mean (±standard deviation, SD) of at least three independent experiments.

**Figure S3**. ^1^H NMR spectrum of cnicin (compound **1**) recorded in CDCl_3_ at 500 MHz.


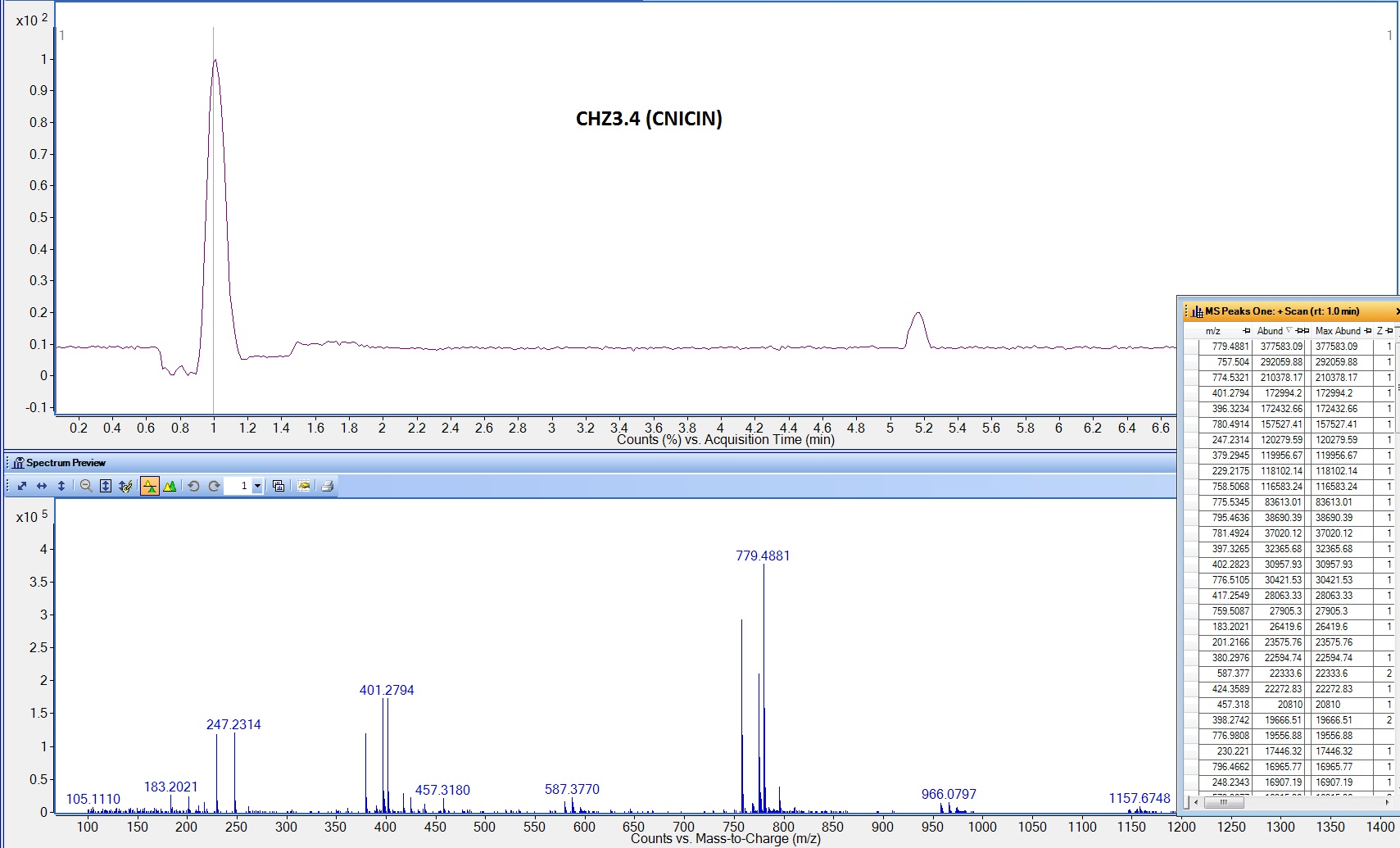


**Figure S4.** ESI MS spectrum of cnicin (compound **1**) recorded in positive modality.

**Figure S5**. ^1^H NMR spectrum of 11β,13-dihydrosalonitenolide (compound **2**) recorded in CDCl_3_ at 500 MHz.


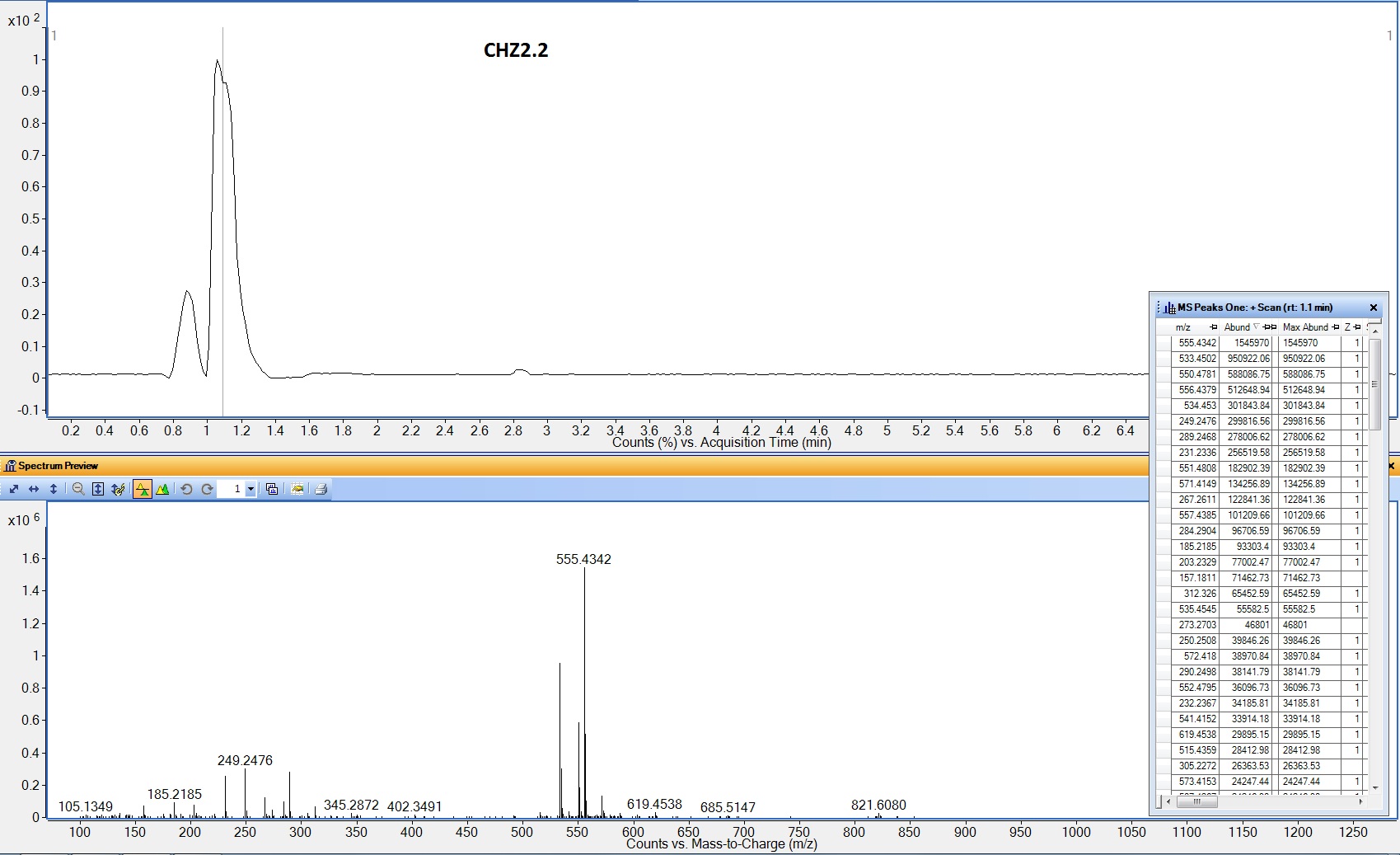


**Figure S6.** ESI MS spectrum of 11β,13-dihydrosalonitenolide (compound **2**) recorded in positive modality.

**Figure S7**. ^1^H NMR spectrum of salonitenolide (compound **3**) recorded in CD_3_OD at 500 MHz.


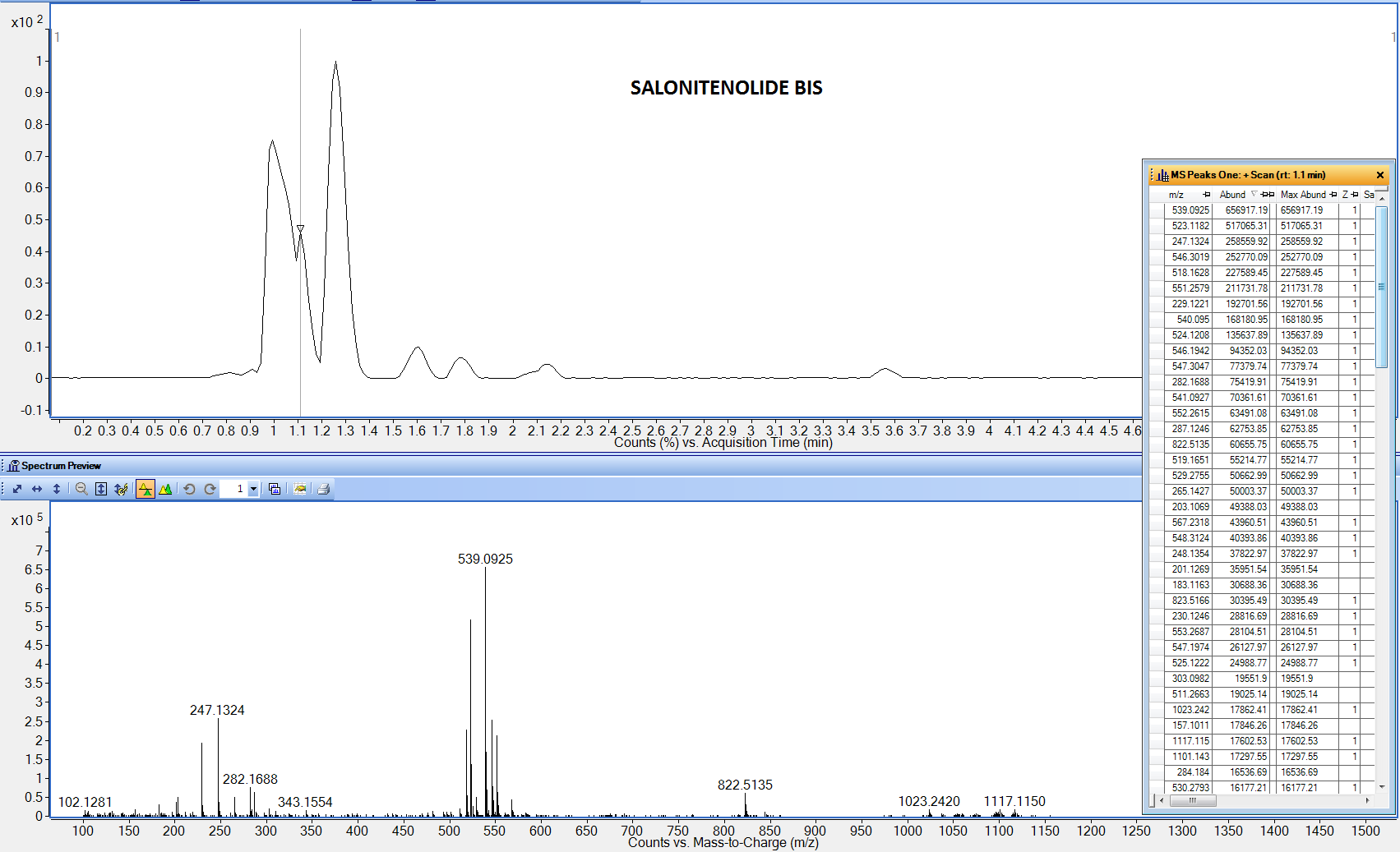


**Figure S8.** ESI MS spectrum of salonitenolide (compound **3**) recorded in positive modality.

**Table S1:** MIC values range of the tested bacterial strains against some antibitics based on CDC web site, CLSI report 2020 and EUCAST web site. The (-) indicates no value were regitired

| MIC_100_ (µg/mL) | | | |
| --- | --- | --- | --- |
|  | **Ampicillin** | **Gentamicin** | **Vancomycin** |
| *S. aureus* ATCC | <=0.06 - >= 4 | <=2 - >= 64 | <=0.5 - 2 |
| *S. aureus* MRSA | - | - | 8 - ≥ 32 |
| *Enterococcus spp.* | <=8 - >=16 | <=0.06 - >= 4 | <=4 - >=32 |
| *E. coli* | 0.03- 0.5 | 0.016 - 32 | - |
| *S. Typhimurium* | 1 - 4 | 0.5 - 2 | - |
|  |  |  |  |
| *Acinetobacter spp.* | <=16 - >= 128 | <=4 - >= 16 | - |

- CLSI. Performance Standards for Antimicrobial Susceptibility Testing. 30th ed. CLSI supplement M100.Wayne, PA: Clinical and Laboratory Standards Institute; 2020.
- <https://www.eucast.org/>
- <https://www.cdc.gov/https://www.eucast.org/>
